# Classifying the diagnosis of study participants in clinical trials: a structured and efficient approach

**DOI:** 10.1186/s41747-020-00169-y

**Published:** 2020-07-17

**Authors:** Tjitske S. R. van Engelen, Maadrika M. N. P. Kanglie, Inge A. H. van den Berk, Merel L. J. Bouwman, Hind J. M. Suhooli, Sascha L. Heckert, Jaap Stoker, Patrick M. M. Bossuyt, Jan M. Prins, For the OPTIMACT Study Group, Jouke Annema, Ludo F. M. Beenen, Inge A. H. van den Berk, Shandra Bipat, Patrick M. M. Bossuyt, Paul Bresser, Marcel Dijkgraaf, Jos Donker, Tjitske S. R. van Engelen, Betty Frankemölle, Maarten Groenink, Suzanne M. R. Hochheimer, Frits Holleman, Dorine Hulzebosch, Maadrika M. N. P. Kanglie, Mitran Keijzers, Ivo van der Lee, Peter Leenhouts, Jan Luitse, Lilian J. Meijboom, Saskia Middeldorp, Alexander Montauban van Swijndregt, Wouter de Monyé, Jacqueline Otker, Milan Ridderikhof, Johannes A. Romijn, Antoinet J. N. Schoonderwoerd, Ralf W. Sprengers, Jaap Stoker, Elizabeth M. Taal, Michiel Winter, Jan M. Prins

**Affiliations:** 1 Department of Internal Medicine, Center for Experimental and Molecular Medicine, Amsterdam UMC, University of Amsterdam, Meibergdreef 9, 1105 AZ, Room: T1-0-240, Amsterdam, The Netherlands; 2 Department of Radiology and Nuclear Medicine, Amsterdam UMC, University of Amsterdam, Meibergdreef 9, 1105 AZ Amsterdam, The Netherlands; 3 Department of Clinical Epidemiology, Biostatistics and Bioinformatics, Amsterdam UMC, University of Amsterdam, Meibergdreef 9, 1105 AZ Amsterdam, The Netherlands; 4 Department of Internal Medicine, Division of Infectious Diseases, Amsterdam UMC, University of Amsterdam, Meibergdreef 9, 1105 AZ Amsterdam, The Netherlands

**Keywords:** Emergency service (hospital), Methods, Observer variation, Radiography (thoracic), Tomography x-ray, computed

## Abstract

**Background:**

A challenge in imaging research is a diagnostic classification of study participants. We hypothesised that a structured approach would be efficient and that classification by medical students, residents, and an expert panel whenever necessary would be as valid as classification of all patients by experts.

**Methods:**

OPTIMACT is a randomised trial designed to evaluate the effectiveness of replacing chest x-ray for ultra-low-dose chest computed tomography (CT) at the emergency department. We developed a handbook with diagnostic guidelines and randomly selected 240 cases from 2,418 participants enrolled in OPTIMACT. Each case was independently classified by two medical students and, if they disagreed, by the students and a resident in a consensus meeting. Cases without consensus and cases classified as complex were assessed by a panel of medical specialists. To evaluate the validity, 60 randomly selected cases not referred to the panel by the students and the residents were reassessed by the specialists.

**Results:**

Overall, the students and, if necessary, residents were able to assign a diagnosis in 183 of the 240 cases (76% concordance; 95% confidence interval [CI] 71–82%). We observed agreement between students and residents *versus* medical specialists in 50/60 cases (83% concordance; 95% CI 74–93%).

**Conclusions:**

A structured approach in which study participants are assigned diagnostic labels by assessors with increasing levels of medical experience was an efficient and valid classification method, limiting the workload for medical specialists. We presented a viable option for classifying study participants in large-scale imaging trials (Netherlands National Trial Register number NTR6163).

## Key points

- Valid diagnostic classification of study participants is a prerequisite for imaging research.
- A structured approach, using the expertise of students, residents, and medical specialists, was an efficient classification method of patients, limiting the workload for medical specialists.
- Medical students and residents could classify the diagnosis of patients suspected of pulmonary disease at the emergency department with high validity, as compared to a panel of medical specialists.

## Background

Valid and reliable classification of the clinical diagnosis of study participants is a prerequisite for the evaluation of new and existing imaging strategies. To conduct such studies, ideally, a single reference standard evaluation is available for disease classification [1]. If such a reference standard evaluation is not available, alternative classification methods include the use of a clinical reference standard, such as a panel-based diagnosis [2]. Relying on an expert panel of medical specialists who retrospectively evaluate the available test results and additional findings to classify study participants in a consensus procedure, is regarded as an acceptable approach [3]. Unfortunately, classification by medical specialists of large numbers of patients is often not feasible, due to budget and manpower restraints.

To obtain a valid and cost-efficient classification of patients suspected of pulmonary disease, we developed a structured approach that involves medical students, residents, an expert panel, and a detailed handbook. We developed diagnostic guidelines that combine multiple test results via predefined deterministic rules. We evaluated this diagnostic handbook in a diagnostic study on chest imaging. The OPTIMACT trial (OPTimal IMAging strategy in patients suspected of non-traumatic pulmonary disease at the ED: chest x-ray or ultra-low-dose (ULD) chest CT) is designed to evaluate the effectiveness of replacing chest x-ray for ULD chest CT in the diagnostic work-up of patients suspected of non-traumatic pulmonary disease at the emergency department (ED) [4].

We hypothesised that our structured approach using a carefully developed reference standard for diagnostic classification would be efficient and that such classification by a team of medical students, residents, and an expert panel would be valid.

## Methods

### Subjects

This study was performed within the framework of the OPTIMACT randomised controlled trial (RCT). Specifics of the study protocol can be found elsewhere [4]. Briefly, the OPTIMACT trial is a multicentre, pragmatic RCT with a non-inferiority design to evaluate the effectiveness of replacing chest x-ray for ULD chest CT in the diagnostic work-up of patients suspected of non-traumatic pulmonary disease at the ED.

For the evaluation of our classification strategy, we took a stratified, random subset of 240 OPTIMACT participants (10%), using a random number generator. The size of this subset was based on comparable evaluations performed earlier [5]. We ensured a 1:1 ratio of chest x-ray *versus* ULD chest CT (120/120) and a 2:1 ratio of participants enrolled at the two participating hospitals (160/80), matching the distribution in the OPTIMACT cohort.

### Diagnostic handbook

The research team, consisting of a chest radiologist, an internist, a pulmonologist, and a cardiologist, carefully developed a handbook consisting of diagnostic classification rules (Supplemental material 1). We defined 26 thoracic diagnostic labels for adults suspected of non-traumatic pulmonary disease at the ED. These diagnostic labels can be divided into five diagnostic categories: respiratory tract infections, other pulmonary diseases, heart diseases, vascular diseases, nodules, and tumours. Each diagnostic label was based on recent diagnostic guidelines and defined by either a reference standard (*e.g.*, pneumothorax) or a composite reference (*e.g.*, pneumonia). For patients not fulfilling one of these 26 definite diagnostic labels, we defined six additional diagnostic categories: thoracic pain of unknown origin, dyspnoea of unknown origin, fever of unknown origin, other thoracic pathology, extrathoracic pathology, and no pathology. We devised decision rules to define cases with signs of complexity (such as empyema, suspicion of a radiation pneumonitis, or a possible primary episode of interstitial lung disease).

### Assessors

In a pilot study in randomly selected OPTIMACT trial participants, we found that medical students using our diagnostic handbook agreed in only 32 out of 75 cases (43%). We therefore devised a strategy where cases in which students disagreed on the diagnosis were additionally assessed by a resident. If the medical students and the resident could not reach consensus, a final assessment was made by an expert panel of medical specialists.

The students were paired from a pool of six medical students. All students had a Bachelor’s Degree in Medicine. The residents (either T.v.E. or M.K.) had at least 1 year of clinical experience as a physician. The expert panel consisted of four medical specialists: a chest radiologist, an internist, a pulmonologist, and a cardiologist. All experts had at least 3 years of experience in their field. None of them was a member of the research team. All observers were trained in the use of the diagnostic handbook using case vignettes.

### Study design

All cases were assessed in a structured approach based on a review of all clinical, radiological, and microbiological data available after 28 days of follow-up. Study participants could have more than one diagnosis; we did not make a distinction between primary and secondary diagnoses. Only the clinical condition that was the reason for the current ED presentation was labelled.

**Fig. 1:**
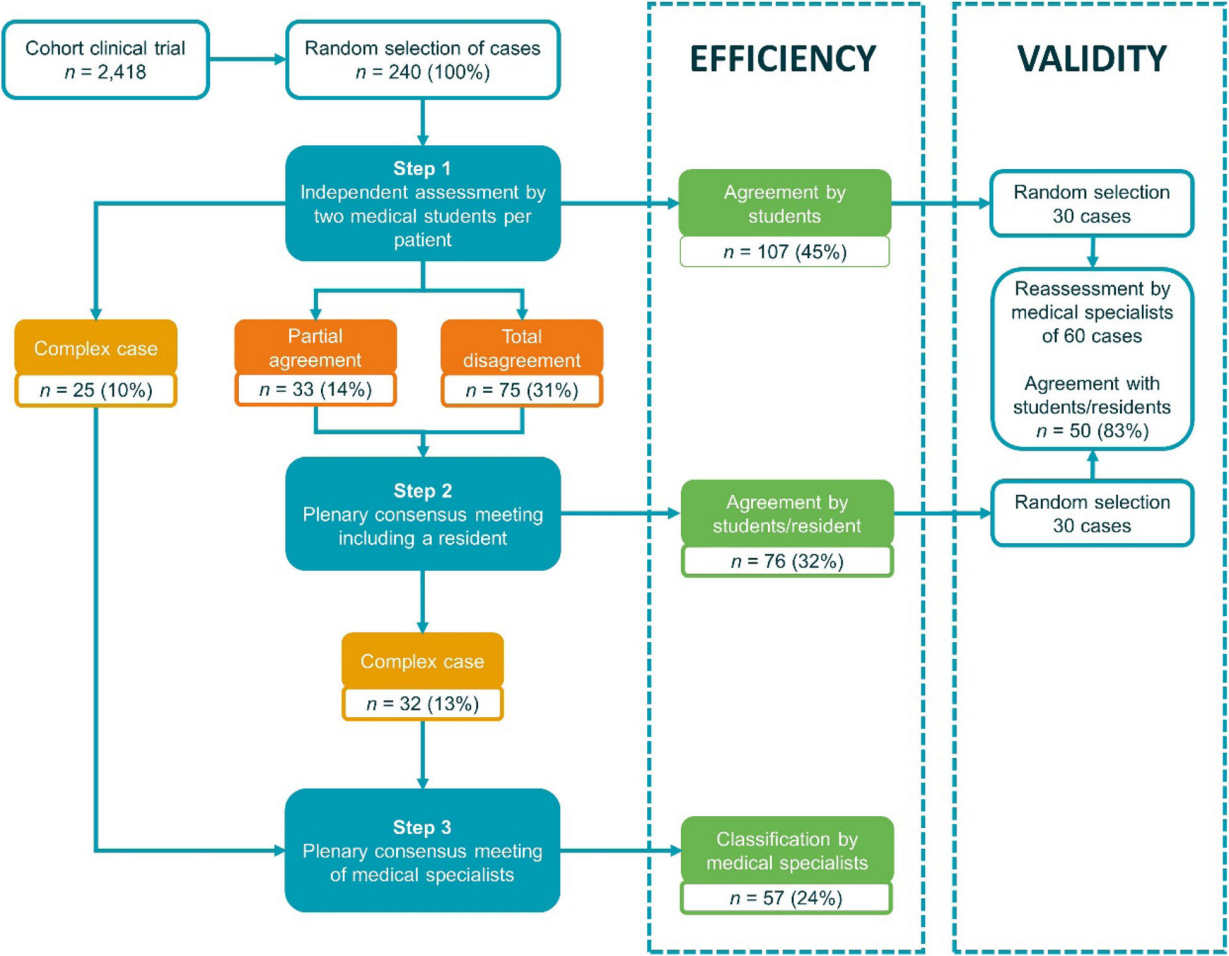
Study design and main results

Each case was independently assessed by two medical students using data present in the electronic health record (EHR) (step 1) (Fig. 1). Cases meeting the predefined criteria of complexity were directly referred to the expert panel if agreed upon by the students. If there was total agreement on all diagnostic labels, the participant was classified accordingly. If not, the case was additionally assessed by a resident who did not know the assessment of the students. The two students and the resident then discussed the case in a consensus meeting (step 2). During the meeting, a chair (a member of the research team) introduced the case, led the discussion, kept track of time, and ensured consistency of assessments by keeping a log. If consensus was reached within 10 min, the case was classified accordingly. If consensus could not be reached, or the case was deemed too complex, it was referred to the expert panel (step 3).

Two members of the expert panel (the internist and the pulmonologist) were unaware of previous assessments and received paper vignettes, which they assessed individually. The cardiologist only received those cases where at least one cardiologic label was assigned or the additional diagnostic category “thoracic pain of unknown origin” or “other thoracic pathology” was assigned. The cardiologist then provided feedback in writing. If all panel members who were involved on a paper case vignette agreed on the diagnostic label(s), the participant was classified accordingly. If not, three members of the research team T.v.E., M.K., J.P. assessed the case for procedural errors. All remaining disagreements were discussed in a plenary meeting by the internist, pulmonologist, and chest radiologist, until consensus was reached. It was the role of the chest radiologist to reassess images on the spot, when deemed necessary. The meeting was chaired as previously described.

To validate the diagnoses assigned by students and residents, we randomly selected 30 cases classified by students and 30 cases classified during the consensus meeting of students and a resident. These 60 cases were reassessed and classified by the expert panel in a similar method as previously described.

### Outcome variables

The primary outcomes of the evaluation were the efficiency of the structured approach and the validity of classifications by the medical students and residents. Efficiency was defined as the percentage of participants to whom a diagnosis could be assigned by the students and residents without evaluation by the expert panel. As the OPTIMACT trial focuses on thoracic pathology, disagreements on extrathoracic pathology were ignored. To illustrate the efficiency of our method, we calculated the reduction in working hours needed by medical specialists to classify the entire OPTIMACT study group of 2,418 patients, which was set against the hours needed by students and residents to classify patients.

We also evaluated the validity of classifications by the medical students and residents, defined as an agreement between their classification and the classification by the expert panel. Possible outcomes were total agreement (defined as agreement on all diagnostic labels), partial agreement (agreement on at least one, but not all diagnostic labels), or total disagreement. The agreement includes cases where disagreement on the diagnosis was based on procedural errors or on discordance on labels from the additional diagnostic categories only. An additional goal was to get a qualitative impression of the differences between classifications done by students and residents *versus* the expert panel. Therefore, the partial agreement and total disagreement cases were studied in detail.

In addition, we evaluated the reasons for referral of a case to the expert panel, consistency of the diagnostic handbook (defined as an overall inter-observer agreement between the students), reasons for disagreement between the students, inter-observer agreement between students for specific diagnostic labels, and classification by and inter-observer agreement between members of the expert panel.

### Statistical analysis

Percentages were calculated with their 95% confidence interval (CI) [6]. An agreement percentage ≥ 80% was regarded as an acceptable inter-observer agreement. To correct for agreement by chance on diagnostic labels, Cohen’s kappa (*κ*) statistics were calculated with a 95% CI [7]. We categorised *κ* agreement as very good (0.81–1.00), good (0.61–0.80), moderate (0.41–0.60), fair (0.21–0.40), or poor (< 0.20) [8, 9]. Data were analysed using SPSS 24 (2019, IBM Software, Armonk, NY, USA).

## Results

### Demographics

The 240 cases that were randomly selected from the 2,418 patients enrolled in the OPTIMACT trial did not differ from the non-selected cases in age, gender, and comorbidities, based on a preliminary analysis prior to data cleaning (data not shown). Follow-up at 28 days was complete for all cases.

### Efficiency of the method

One hundred seven of the 240 participants (45%) could be assigned a diagnosis by the students without additional evaluation and 76 more (32%) by students and residents after a consensus meeting (Fig. 1). This way, only 57 of 240 cases (24%) had to be referred to the experts. There were four cases with disagreement on extrathoracic pathology, which were ignored in this study on thoracic pathology and therefore counted as agreement.

Of the 108 cases discussed in the consensus meeting between students and a resident, 32 cases were referred to the expert panel. The reasons for referral of a case to the expert panel were predefined rules of the diagnostic handbook (*n* = 12), specific questions for the experts (*n* = 10), case complexity (*n* = 9), or because no consensus could be obtained (*n* = 1).

When this method would be applied in the entire OPTIMACT study group of 2,418 patients, we estimate to save a total of 703 h of work by medical specialists. This has to be set against the (less expensive) hours of students and residents, which would approximate 900 h and 286 h, respectively. Calculations can be found in Supplemental material 2.

### Validity

Validation by the expert panel of 60 randomly selected cases resulted in an agreement between students (30 cases) or students and resident (30 cases) *versus* the expert panel for 50 of the 60 cases (83% concordance; 95% CI 74–93%). In 46 cases (77%), there was a total agreement, in 10 cases (17%) partial agreement, and in 4 cases (7%) disagreement on the classification. Further evaluation of the 14 partial and disagreement cases revealed that 4 cases were due to discordance on labels from the additional diagnostic categories only or procedural errors, leaving 10 cases as “true” disagreement.

Of the 30 cases classified by students only there was a total agreement with the expert panel in 27 cases and partial agreement in 3 (Table 1): one case where the students overdiagnosed an upper respiratory tract infection (URTI), one case of demand ischemia which the students labelled as an acute coronary syndrome, and one case where the students deemed a new finding of atrial fibrillation a chance finding, not related to the reason for ED presentation. Of the 30 participants classified during the consensus meeting between students and a resident, there was a total agreement with the expert panel in 19 cases, partial agreement in 7 cases, and disagreement in 4 (Table 2). Among the partial agreement cases (7/30), there was one case where the students and the resident diagnosed a community-acquired pneumonia (CAP) in addition to a pulmonary embolism, whereas the expert panel deemed the infiltrate on the chest x-ray an infarction due to the pulmonary embolism. Furthermore, there were two cases where the students overdiagnosed an URTI and one case where the students deemed a history of lung transplantation not relevant for the current ED presentation. There was one case with discordance due to a procedural error, one case with discordance on the students’ additional diagnostic label thoracic pain of unknown origin, and one case with discordance on the students’ additional diagnostic label extrathoracic pathology. In the four cases of disagreement, there was one case concerning a patient with a pulmonary infection in addition to a urinary tract infection. The chest x-ray described as bronchiolitis, and the students classified this as a lower respiratory tract infection (LRTI), other than CAP. The expert panel reassessed the chest x-ray, found arguments that contradicted the chest x-ray report, and assigned a diagnosis of CAP. There were two cases where the students overdiagnosed an URTI and one case with discordance on the additional diagnostic label thoracic pain of unknown origin.

**Table 1:** Classification by medical students as compared to classification by the expert panel (*n* = 30)

Diagnostic label(s) expert panel	Number of cases	Diagnostic labels(s) by medical students
Agreement	27	
Single label cases	20	
Extrathoracic pathology	6	
Community-acquired pneumonia	4	
Thoracic pain of unknown origin	4	
Influenza A or B	3	
Fever of unknown origin	1	
Healthcare-associated pneumonia	1	
Other lower respiratory tract infection	1	
Multiple label cases	7	
Community-acquired pneumonia, exacerbated chronic obstructive pulmonary disease	2	
Community-acquired pneumonia, influenza A or B	1	
Exacerbated chronic obstructive pulmonary disease, cardiac failure	1	
Influenza A or B, exacerbated asthma	1	
Other lower respiratory tract infection, exacerbated asthma	1	
Thoracic pain of unknown origin, other thoracic pathology	1	
Partial agreement	3	
Single-label cases	1	
Cardiac arrhythmia	1	Cardiac arrhythmia, other upper respiratory tract infection
Multiple label cases	2	
Community-acquired pneumonia, cardiac arrhythmia	1	Community-acquired pneumonia
Influenza A or B, exacerbated chronic obstructive pulmonary disease	1	Influenza A or B, exacerbated chronic obstructive pulmonary disease, acute coronary syndrome with elevated troponin levels
Disagreement	0	

Within categories of agreement, rows are sorted first by prevalence and then alphabetically

**Table 2:** Classification by medical students and a resident as compared to classification by the expert panel (*n* = 30)

Diagnostic label(s) expert panel	Number of cases	Diagnostic labels(s) medical students and resident
Agreement	19	
Single-label cases	8	
Extrathoracic pathology	3	
Healthcare-associated pneumonia	2	
Cardiac failure	1	
Other thoracic pathology	1	
Other upper respiratory tract infection	1	
Multiple label cases	11	
Community-acquired pneumonia, influenza A or B	3	
Community-acquired pneumonia, exacerbated asthma	1	
Community-acquired pneumonia, interstitial lung disease	1	
Community-acquired pneumonia, other thoracic pathology	1	
Cardiac failure, cardiac arrhythmia	1	
Fever of unknown origin, other thoracic pathology	1	
Other lower respiratory tract infection, exacerbated asthma	1	
Other lower respiratory tract infection, exacerbated chronic obstructive pulmonary disease	1	
Sinusitis, exacerbated asthma	1	
Partial agreement	7	
Single-label cases	2	
Extrathoracic pathology	1	Extrathoracic pathology, thoracic pain of unknown origin^a^
Pulmonary embolism	1	Pulmonary embolism, community-acquired pneumonia
Multiple label cases	5	
Exacerbated asthma, exacerbated chronic obstructive pulmonary disease	1	Exacerbated asthma, exacerbated chronic obstructive pulmonary disease, other upper respiratory tract infection
Exacerbated asthma, thoracic pain of unknown origin	1	Exacerbated asthma, other upper respiratory tract infection
Influenza A or B, other thoracic pathology	1	Influenza A or B
Other lower respiratory tract infection, influenza A or B	1	Influenza A or B^a^
Other upper respiratory tract infection	1	Other upper respiratory tract infection, extrathoracic pathology^a^
Disagreement	4	
Single-label cases	3	
Dyspnoea of unknown origin	1	Other upper respiratory tract infection
Fever of unknown origin	1	Other upper respiratory tract infection
Other thoracic pathology	1	Thoracic pain of unknown origin^a^
Multiple label cases	1	
Community-acquired pneumonia, extrathoracic pathology	1	Other lower respiratory tract infection, extrathoracic pathology

Within categories of agreement, rows are sorted first by prevalence and then alphabetically

^a^Disagreement on the diagnosis was based on discordance on a procedural error or labels from the additional diagnostic categories only. These are considered agreement cases

### Overall inter-observer agreement between students

The total agreement was observed for 132 of the 240 participants (55%): 107 (45%) were assigned the same diagnostic label(s) and 25 (10%) were directly referred to the expert panel. Cases where the two students disagreed (108, 45%) were sub-classified as a partial agreement (33, 14%) and total disagreement (75, 31%) (Fig. 1). In 39 of the 75 total disagreement cases, one of the two students deemed the case too complex and initially referred this case to the expert panel. Twenty-one of these 39 cases were classified during the consensus meeting with a resident and could be withheld from referral to the expert panel. Reasons for disagreement between students can be found in Supplemental material 3.

### Inter-observer agreement between students for specific diagnostic labels

A total of 523 labels were assigned to the 240 cases: 336 definite diagnostic labels (concordance 65%) and 187 labels from the additional six diagnostic categories (concordance 61%) (Supplemental Table S1). The most prevalent diagnostic labels were LRTI other than CAP (70/336, concordance 49%; κ 0.43, 95% CI 0.27–0.59), CAP (67/336, concordance 81%; κ 0.78, 95% CI 0.66–0.89), exacerbation chronic obstructive pulmonary disease (COPD) (42/336, concordance 76%; κ 0.74, 95% CI 0.59–0.89), exacerbation asthma (30/336, concordance 73%; κ 0.72, 95% CI 0.53–0.90), and influenza A/B (28/336, concordance 79%; κ 0.77, 95% CI 0.60–0.95). The most prevalent additional diagnostic category was extrathoracic pathology (101/187, concordance 67%; κ 0.59, 95% CI 0.46–0.71). Direct referral to the expert panel was selected 89 times of which 50 selections resulted in 25 agreement cases (concordance 56%).

### Classification by and inter-observer agreement between members of the expert panel

Classification by the expert panel in the 60 validation cases resulted in an agreement between internist, pulmonologist, and cardiologist (if necessary) in 34 cases (concordance 57%, 95% CI 44–69%). In 24 cases (40%), there was a total agreement, in 23 cases (38%) partial agreement, and in 13 cases (22%) disagreement on the classification. Further qualitative evaluation of the 36 partial and disagreement cases revealed that 10 cases were due to discordance on labels from the additional diagnostic categories only or procedural errors, leaving 26 cases as “true” disagreement. Specifics can be found in Supplemental Table S2.

A total of 173 labels were assigned to the 60 validation cases by the expert panel: 119 definite diagnostic labels (concordance 86%) and 57 labels from the additional six diagnostic categories (concordance 70%). Specifics and *κ* values can be found in Supplemental Table S3.

## Discussion

We tested a method for post hoc classification of study participants in large scale radiology trials in a study comparing chest x-ray with ultra-low-dose chest CT. The students and, if necessary, residents were able to assign a diagnosis in 76% of cases with a suspicion of pulmonary disease. Comparing the classification of 60 patients by medical students and residents against the classification of the same patients by a panel of medical specialists resulted in agreement on the clinical diagnosis for 50 of the 60 patients (83% concordance, 95% CI 74–93%). When discrepancies were studied in detail, students classified in particular more less severe diagnoses, such as URTI, that the medical specialist put aside.

The use of a composite reference is a common method for disease classification in large clinical trials. As an example, in a diagnostic accuracy study evaluating imaging strategies for the detection of urgent conditions in patients with acute abdominal pain, a final diagnosis was assigned by an expert panel of two gastrointestinal surgeons and an abdominal radiologist [10]. Laméris et al. described the general methods for diagnosis assignment and listed the panel members in their appendix. Specifics on how the panel was instructed, blinding of members, measures of agreement, and the process of the consensus meeting are not provided in the main study report. Word count limits imposed by journals complicate full and informative reporting of such essential issues, and as a result, methods to achieve panel-based consensus are often not described in studies, precluding reproducibility, and guidance on preferred methodology is lacking [3, 11, 12]. Considering panel-based consensus methods for a trial design might also be discouraged by the time-consuming process of panel-based diagnosis [13, 14].

If the methodology of panel-based diagnosis ís described, agreement regarding diagnosis assignment varies. For instance, Klein Klouwenberg et al. studied the inter-observer agreement in 168 patients who experienced an infectious episode in the intensive care unit [5]. Each case was independently assessed by two research physicians working at least 6 months on the project, who scored the source of infection using a composite reference standard. The agreement was 89% and 69% for a partial and complete diagnostic match, respectively. In addition, the authors found varying agreement from 35 to 97% within specific diagnostic subgroups, with 89% concordance for CAP [5]. A study that investigated the effects of an imperfect reference standard on study outcome suggests that even an almost perfect reference standard can lead to estimates with considerable error [15].

In the present study, total agreement after the first assessment by medical students was observed for 132 of the 240 participants (55%). Agreement among prevalent diagnoses varied from 49% (LRTI other than CAP) to 81% (CAP), indicating that assessment of predefined cases by medical students may lead to similar agreement rates as compared to the assessment by well-trained physicians in Klein Klouwenberg’s study [5]. Notably, after individual assessment of paper vignettes by the internist, pulmonologist, and, if necessary, cardiologist, the members of the expert panel reached a mutual agreement in only 34 of 60 cases (57% concordance; 95% CI 44–69%). Furthermore, an inter-observer agreement between members of the expert panel also varied (*e.g.*, concordance for LRTI other than CAP was 55%, for cardiac failure 75%, and for CAP 100%). Our results underscore the necessity for consensus diagnosis as an individual assessment of cases leads to considerable disagreement—not only among students or residents, but also among fully trained and experienced medical specialists. Our study differs from previous work as this additional assessment was performed if there was disagreement after the first assessment.

The validation process of the study has the intrinsic difficulty that the student and residents followed the guidelines presented in the structured handbook, whereas the expert panel also carries its years’ long experience. The qualitative evaluation of the 60 validation cases showed that differences mainly occurred in less severe diagnoses, such as an URTI. For this specific diagnostic label, students may conclude based on the strict guidelines of the handbook that a patient suffered from an URTI, whereas the medical experts may attach little importance to this if the more clinically relevant diagnosis of heart failure is also present. As shown, comparing the classification of 60 patients by medical students and residents with that of the panel of medical specialists resulted in agreement on the clinical diagnosis for 50 of the 60 patients. If the URTIs are not counted as disagreement, there would have been agreement for 55 of the 60 cases (92% concordance, 95% CI 85–99%). We conclude that students formally classify more less severe diagnoses such as URTI that a medical specialist would put aside.

What is undisputable is that the presented method is efficient, with classification by medical experts limited to 24% of the study population and further shown by the estimated reduction of 703 h of work by medical specialists in this RCT.

The development and use of the diagnostic handbook can be considered one of the strengths of our approach. All members of the classification teams (*i.e.*, students, residents, and the expert panel) used the same structured diagnostic labels as a guideline for their assessment. The provision of such a reference will add to the consistency of disease classification in all steps of the process. In addition, we structured the consensus meetings, including blinding of the attendants for each other’s assessments, an independent technical chairman, and keeping a research log. This process and its detailed reporting further contributed to consistency.

A limitation of the study is that the diagnosis of the expert panel was considered the “true” diagnosis. When assessing disagreement cases in a qualitative matter, we observed several “overdiagnoses” of an URTI by the students and residents. However, an alternative interpretation might be that there is an underdiagnosis of these URTIs by experts. Furthermore, the results of the validity of this study cannot be generalised to other conditions and settings. The participating medical students, residents, and medical specialists were all trained in a Dutch healthcare setting. Prevalence and degree of exposure to the spectrum of medical conditions presenting at the ED likely influenced their assessment of the cases. However, our structure was designed to include general high-volume diseases and addresses a common problem: how to classify patients in large scale clinical trials? This methodology to classify patients could be used in other settings, after the development of a local guideline with diagnostic criteria and an internal validation of these guidelines.

In conclusion, we developed a valid and efficient method to classify the diagnosis of patients suspected of pulmonary disease at the ED, using the expertise of medical students, residents, and medical specialists. We hope that the description of the methods used in our study will inspire other study authors to be equally informative in terms of their description of methods. We believe that the structured approach presented here offers a viable option for classifying study participants in large-scale clinical trials.

## Supplementary information

**Additional file 1: Supplemental material 1.** Diagnostic handbook. **Supplemental material 2.** Calculations of the reduction of working hours for medical specialists set against the hours of students and residents. **Supplemental material 3.** Reasons for disagreement between students. **Supplemental material 4.** Members of the OPTIMACT Study Group. **Supplemental Table S1**. Inter-observer agreement between students for specific diagnostic labels in 240 cases. **Supplemental Table S2**. Classification by the expert panel in 60 validation cases. **Supplemental Table S3**. Inter-observer agreement between members of the expert panel for specific diagnostic labels in 60 validation cases.

## Data Availability

The datasets used and/or analysed during the current study are available from the corresponding author on reasonable request.

## Abbreviations

CAP: Community-acquired pneumonia
CI: Confidence interval
ED: Emergency department
LRTI: Lower respiratory tract infection
RCT: Randomised controlled trial
ULD: Ultra-low-dose
URTI: Upper respiratory tract infection
