# Supplementary material for: Classifying the diagnosis of study participants in clinical trials: a structured and efficient approach
Source: Eur Radiol Exp. 2020 Jul 17;4:44. doi: 10.1186/s41747-020-00169-y (PMC7366867; doi:10.1186/s41747-020-00169-y)
Supplement: Supplementary file 1 — Additional file 1: Supplemental material 1. Diagnostic handbook. Supplemental material 2. Calculations of the reduction of working hours for medical specialists set against the hours of students and residents. Supplemental material 3. Reasons for disagreement between students. Supplemental material 4. Members of the OPTIMACT Study Group. Supplemental Table S1. Inter-observer agreement between students for specific diagnostic labels in 240 cases. Supplemental Table S2. Classification by the expert panel in 60 validation cases. Supplemental Table S3. Inter-observer agreement between members of the expert panel for specific diagnostic labels in 60 validation cases. [file 41747_2020_169_MOESM1_ESM.docx]

**ELECTRONIC SUPPLEMENTARY MATERIAL**

Classifying the diagnosis of study participants in clinical trials: a structured and efficient approach

Tjitske S.R. van Engelen, Maadrika M.N.P. Kanglie, Inge A.H. van den Berk, Merel L.J. Bouwman, Hind J.M. Suhooli, Sascha L. Heckert, Jaap Stoker, Patrick M.M. Bossuyt, Jan M. Prins*

**Supplemental material 1.** Diagnostic handbook

**Supplemental material 2.** Calculations of the reduction of working hours for medical specialists set against the hours of students and residents

**Supplemental material 3.** Reasons for disagreement between students

**Supplemental material 4.** Members of the OPTIMACT Study Group

**Supplemental Table S1**. Inter-observer agreement between students for specific diagnostic labels in 240 cases

**Supplemental Table S2**. Classification by the expert panel in 60 validation cases

**Supplemental Table** **S3**. Inter-observer agreement between members of the expert panel for specific diagnostic labels in 60 validation cases

The authors provide this supplementary material as additional information.

**Supplemental material 1.** Diagnostic handbook

GUIDELINES

1. Start the assessment of each patient by following the flowchart (next page).
2. More than one diagnosis can be applicable per patient. Please go through the entire handbook for each patient, irrespective of already assigned diagnostic labels.
3. Refer a case to the Adjudication Committee when two or more labels “…of unknown origin” are assigned (i.e. “Thoracic pain of unknown origin”, “Dyspnea of unknown origin”, or “Fever of unknown origin”).
4. If a patient has new complaints after ED discharge but within 28 days of follow-up, please review critically if these complaints correspond to a diagnosis that was already present during ED presentation. If not, do not label this diagnosis. To clarify: the diagnostic labels are a reflection of the diagnoses at ED presentation and not meant for new problems that emerge during hospitalization or after hospital discharge.
5. If you are confronted with a very complex case (for example a patient with many concurrent problems, complex comorbidities or unclear documentation), you are encouraged to refer this case to the Adjudication Committee even if the guidelines in the handbook do not state so.
6. Use your common sense. Using this handbook allows you to diagnose patients into several predefined categories. However, sometimes this is not straightforward. If the guidelines lead you to a diagnostic label that you do not support, either choose the label that you find most suitable or refer the case to the Adjudication Committee.
7. If follow-up data is missing (i.e. a patient has been transferred to another hospital but the transfer correspondence has not been uploaded yet), please indicate so on the assessment form.

LIST WITH DIAGNOSTIC LABELS

Respiratory tract infections .

1. Community-acquired pneumonia
2. Healthcare-associated pneumonia
3. Aspiration pneumonia
4. Radiation pneumonitis
5. Other lower respiratory tract infection (bronchitis, bronchiolitis)
6. Influenza A/B
7. Sinusitis
8. Other upper respiratory tract infection

Other pulmonary diseases

1. Exacerbation asthma
2. Exacerbation chronic obstructive pulmonary disease
3. Exacerbation cystic fibrosis
4. Pleural effusion or empyema
5. Atelectasis
6. Pleuritis sicca
7. Pneumothorax
8. Interstitial lung disease
9. Acute Chest Cyndrome

Heart diseases

1. Acute coronary syndrome with elevated troponin levels
2. Acute coronary syndrome without elevated troponin levels
3. Stable angina pectoris
4. Cardiac failure
5. Pericarditis
6. Cardiac arrhythmia

Vascular diagnosis

1. Pulmonary embolism

Nodules and tumors

1. Lung cancer and pulmonary metastases
2. Mediastinal tumor

Other diagnostic labels

1. Thoracic pain of unknown origin
2. Dyspnea of unknown origin
3. Fever of unknown origin
4. Other thoracic pathology
5. Extrathoracic pathology
6. No pathology

DIAGNOSTIC LABELS: WHEN TO ASSIGN THEM

**1. Community-acquired pneumonia (CAP)**

See flowchart.

*References: [1-3].*

**2. Healthcare-associated pneumonia (HCAP)**

See flowchart.

*References: [1-3].*

**3. Aspiration pneumonia**

See flowchart.

*References: [1-3].*

**4. Radiation pneumonitis**

When a radiation pneumonitis is suspected, the case should be sent to the Adjudication Committee.

**5. Other lower respiratory tract infection** (LRTI; bronchitis, bronchiolitis)

See flowchart.

NB: no distinction is made between the two diagnoses bronchitis and bronchiolitis

*References: [1-3].*

**6. Influenza A/B**

See flowchart.

*References: [1-3].*

**7. Sinusitis**

See flowchart.

NB. When a CAP is already diagnosed, this diagnostic label should not be assigned.

*References: [1-4].*

**8. Other upper respiratory tract infections**

See flowchart.

NB. When a CAP is already diagnosed, this diagnostic label should not be assigned.

*References: [1-3].*

**9. Exacerbation asthma**

Prerequisite: the patient has a medical history of asthma.

*Exacerbation asthma*

An acute or sub-acute exacerbation of the symptoms the patient normally experiences:

- Patient has at least one of the following: worsening in shortness of breath, wheezing or “chest tightness” AND
- Additional treatment was started.

Exceptions:

- All asthma patients with a pneumonia or other LRTI, should be labelled “Exacerbation asthma” as well as “Pneumonia” or “Other LRTI”. That is, if the patient has a medical history of asthma and presents at the ED with pneumonia, he or she should automatically be labelled both “Pneumonia” and “Exacerbation asthma”, irrespectively of the treatment that might or might not be started for the exacerbation. However, this does not apply for concurrent sinusitis or other upper respiratory tract infection. When a patient has an exacerbation of asthma and sinusitis or other upper respiratory tract infection, additional treatment for the exacerbation should have been started in order to assign both diagnostic labels.
- This was a first episode of asthma. This case should be referred to the Adjudication Committee.
- If the patient has a medical history of both asthma and chronic obstructive pulmonary disease (COPD), the case should be referred to the Adjudication Committee.

*References: [5]*

**10. Exacerbation chronic obstructive pulmonary disease (COPD)**

Prerequisite: the patient has a medical history of COPD.

*Exacerbation COPD*

An acute or sub-acute exacerbation of the symptoms the patient normally experiences:

1. Patient has at least one of the following: worsening of dyspnea, wheezing, cough, or sputum production, or more purulent sputum AND
2. Additional treatment was started. NB: nebulized β2-sympathomimetics are also considered treatment.

Exceptions:

- All COPD patients with a pneumonia or other LRTI, should be labelled “Exacerbation COPD” as well as “Pneumonia” or “Other LRTI”. That is, if the patient has a medical history of COPD and presents at the ED with pneumonia, he or she should automatically be labelled both “Pneumonia” and “Exacerbation COPD”, irrespectively of the treatment that might or might not be started for the exacerbation. However, this does not apply for concurrent sinusitis or other upper respiratory tract infection. When a patient has an exacerbation of COPD and sinusitis or other upper respiratory tract infection, additional treatment for the exacerbation should have been started in order to assign both diagnostic labels.
- This was a first episode of COPD. This case should be referred to the Adjudication Committee.
- If the patient has a medical history of both asthma and COPD, the case should be referred to the Adjudication Committee.

*References: [6, 7]*

**11. Exacerbation cystic fibrosis (CF)**

Prerequisite: the patient has a medical history of CF.

*Exacerbation CF*

An acute or sub-acute exacerbation of the symptoms the patient normally experiences:

1. Patient has at least one of the following: worsening of dyspnea, hemoptysis, cough or wheezing, or more mucus production or more purulent sputum [8] AND
2. Additional treatment was started.

Exception:

- All CF patients with a pneumonia or other LRTI, should be labelled “Exacerbation CF” as well as “Pneumonia” or “Other LRTI”. That is, if the patient has a medical history of CF and presents at the ED with pneumonia, he or she should automatically be labelled both “Pneumonia” and “Exacerbation CF”, irrespectively of the treatment that might or might not be started for the exacerbation. However, this does not apply for concurrent sinusitis or other upper respiratory tract infection. When a patient has an exacerbation of CF and sinusitis or other upper respiratory tract infection, additional treatment for the exacerbation should have been started in order to assign both diagnostic labels.

*References: [8]*

**12. Pleural effusion or empyema**

Please consider the following three options:

1. The radiology report describes abundant pleural effusion: this case should be referred to the Adjudication Committee.
2. The radiology report describes slight or moderate pleural effusion and there is no conclusive other diagnosis: this case should be referred to the Adjudication Committee.
3. The radiology report describes slight or moderate pleural effusion and there is another conclusive diagnosis (pneumonia, heart failure, or lung tumor): the label pleural effusion is not assigned and only the conclusive diagnosis is labelled.

*References: [9, 10]*

**13. Atelectasis**

Please consider the following two options:

1. The radiology report concludes lobar or segmental atelectasis: this case should be referred to the Adjudication Committee.
2. The radiology report concludes linear (plate, band, discoid, subsegmental) atelectasis: this case should not be referred to the Adjudication Committee and should not be labelled “Atelectasis”.

Exception:

- In case of pre-existing lobar or segmental atelectasis, the case does not need to be referred to the Adjudication Committee and should not be labelled “Atelectasis”.

**14. Pleuritis sicca**

Prerequisite: there are no radiologic findings.

Patient is in pain; mostly related to breathing. The physical examination describes “pleural rub”. Specific echographic finding: irregular visceral pleura. In case of pain without pleural rub or a specific echographic finding: assign the diagnostic label “Thoracic pain of unknown origin”.

**15. Pneumothorax**

The radiology report confirms a pneumothorax.

*References: [11]*

**16. Interstitial Lung Disease**

Prerequisite: the patient has a medical history of lung fibrosis or interstitial lung disease (ILD), this had worsened and/or was relevant at ED presentation and (additional) treatment was started for the ILD.

Exception:

- This was a first episode or a suspicion of a first episode of ILD. This case should be referred to the Adjudication Committee.

Examples of ILD that can be found in a patient’s medical history:

- Sarcoidosis
- Usual interstitial pneumonia (UIP)
- Non-specific interstitial pneumonia (NSIP)
- Extrinsic allergic alveolitis (EAA)
- Organizing pneumonia
- Pigeon breeder's lung, mushroom worker’s lung, cheese-washer’s lung, farmer’s lung, asbestosis
- Drug-induced pneumonitis or drug-induced lung fibrosis (due to amiodarone, nitrofurantoin, methothraxate)
- ILD within the context of systemic diseases like rheumatoid arthritis and scleroderma.

*References: [12]*

**17. Acute Chest Syndrome**

Please assign this diagnostic label when the following criteria apply:

- The patient has a medical history of sickle cell anemia AND
- The radiology report confirms a consolidation AND
- There is at least one of the following symptoms present: fever (temperature ≥38.5° Celsius), tachypnea (>25/min), intercostal retractions, “nasal flaring”, use of accessory muscles during breathing, chest pain, cough, bronchial breath sounds, >2% decrease in SpO2 (O2 saturation) of a documented steady-state value at room air (FiO2=0.21), PaO2 < 60mmHg/8 kPa.

NB: Do not assign the diagnostic labels “Acute Chest Syndrome” and pneumonia to the same patient. It is difficult to make this clinical distinction. Therefore only this diagnostic label (Acute Chest Syndrome) must be assigned to such cases.

*References: [13, 14]*

**18. Acute Coronary Syndrome with elevated troponin levels**

Includes all patients with unstable ischemic heart disease, including non-ST-elevation myocardial infarction (NSTEMI), ST-elevation myocardial infarction (STEMI) and unstable angina pectoris.

Please assign this diagnostic label when the following criteria apply:

- The patient has a history of ischemic heart disease and experiences in rest or during limited exercise chest pain (with radiation to neck, jaw, left arm), and/or less typical symptoms: dyspnea, nausea/vomiting, fatigue, palpitations, syncope AND
- At Academic Medical Center: elevated troponin levels: troponin levels >0.05µg/L at ED presentation, or >50% increase of the second troponin measurement compared to troponin levels at ED presentation OR
- At Spaarne Gasthuis: elevated troponin levels: troponin levels ≥70ng/L at ED presentation, or >100ng/L increase of the second troponin measurement compared to troponin levels at ED presentation.

NB: demand ischemia or type 2 ischemia should be labeled “Other thoracic pathology”.

*References: [15, 16]*

**19. Acute Coronary Syndrome without elevated troponin levels**

Includes all other patients with unstable ischemic heart disease, including non-ST-elevation myocardial infarction (NSTEMI), ST-elevation myocardial infarction (STEMI) and unstable angina pectoris.

Please assign this diagnostic label when the following criteria apply:

- The patient has a history of ischemic heart disease and experience in rest or during limited exercise chest pain (with radiation to neck, jaw, left arm), and/or less typical symptoms: dyspnea, nausea/vomiting, fatigue, palpitations, syncope

*References: [15, 16]*

**20. Stable angina pectoris**

The patient has classical symptoms of angina pectoris:

- Retrosternal complaints
- Complaints provoked by exercise or emotion
- Relief of complaints by rest or nitroglycerine

Exception:

- When “stable angina pectoris” is not explicitly mentioned in the ED report, but treatment with isosorbide mononitrate was started, strongly consider this diagnostic label. When in doubt, refer this case to the Adjudication Committee.

*References: [17]*

**21. Cardiac failure**

The patient has at least one of the following symptoms:

- A medical history of coronary artery disease, arterial hypertension, exposure to cardiotoxic drugs or radiation, usage of diuretics, orthopnea or paroxysmal nocturnal dyspnea.
- A physical examination that reveals rales, bilateral edema of the ankles, heart murmur, elevated central venous pressure.
- An electrocardiogram (ECG) that shows widened QRS and / or pathologic Q-waves.

AND at least one of the following:

- At Academic Medical Center: N-terminal pro b-type natriuretic peptide (NT-proBNP) ≥ 300 pg/mL OR at Spaarne Gasthuis: B-type natriuretic peptide (BNP) ≥ 28.9 pmol/L.
- Abnormal echocardiography at ED presentation. If echocardiography is normal at ED presentation, or unchanged compared to pre-existent abnormalities, there is no cardiac failure.

AND:

- Treatment was started, for instance diuretics were given or the dosage of preexisting diuretics was increased.

Exception:

- When there is no NT-proBNP or BNP measurement done during ED presentation, but there is a very high clinical suspicion of cardiac failure and treatment for cardiac failure was started, strongly consider this diagnostic label. When in doubt, refer this case to the Adjudication Committee.
- Review critically if the patient indeed has the abovementioned symptoms due to cardiac failure or that symptoms are due to an underlying non-cardiac disease, such as renal asthma. The latter should be assigned the diagnostic label “Other thoracic pathology”.

*References: [18, 19]*

**22. Pericarditis**

Please assign this diagnostic label when at least 2 of the following symptoms are present:

- Chest pain
- Pericardial rub
- Diffuse ST elevation of PR depression on ECG
- Pericardial effusion on medical imaging

Exceptions:

- The patient has only one of the required symptoms, but has additional characteristics. Additional characteristics are elevated levels of C-reactive protein (CRP) or erythrocyte sedimentation rate (ESR), or signs of inflammation on magnetic resonance imaging (MRI) or positron emission tomography (PET)-scan. The additional characteristics can be a sign of complexity of the case. Please refer this case to the Adjudication Committee.
- The ED report or follow-up information states “perimyocarditis”. Please refer this case to the Adjudication Committee.

*References: [1, 20]*

**23. Cardiac arrhythmia**

Prerequisite: the complaints at ED presentation are due to this episode of cardiac arrhythmia and other causes have been excluded (i.e. acute coronary syndrome, pulmonary embolism, infection etc.). Cardiac arrhythmia may be indicated in the EHR as “atrial flutter”, “atrial fibrillation” or “AV-block”.

Exception:

- When the ED report or follow-up information states “sinus tachycardia”, please do not assign this diagnostic label. A sinus tachycardia could be due to an underlying cause such as infection.

**24. Pulmonary embolism**

This label can be assigned in two situations:

1. CT-Angiography was positive for pulmonary embolism.
2. No CT-Angiography was performed, but the patient did have:

a. Dyspnea AND

b. Elevated D-dimer AND

c. A first presentation of an echographically confirmed deep vein thrombosis AND

e. No other abnormality was found on CR-thorax or ULD CT.

NB: when in doubt, refer this case to the Adjudication Committee.

NB: a deep vein thrombosis should not be given a diagnostic label in itself.

*References: [21, 22]*

**25. Lung cancer and pulmonary metastases**

This label can be assigned in two situations:

1. Pathology has confirmed lung cancer (lung cancer)
2. Growth of a pulmonary nodus or a new nodus when the patient has a known malignancy confirmed by pathology (pulmonary metastases)

NB: if there is only a clinical suspicion of lung cancer or pulmonary metastases and/or a (PET-)CT or MRI shows signs of lung cancer or pulmonary metastases, please refer this case to the Adjudication Committee.

*References: [23]*

**26. Mediastinal tumor**

This label can be assigned in two situations:

1. The radiology report describes a mediastinal tumor (teratoma, thymoma, thyroid, lymphoma) AND pathology has confirmed a mediastinal tumor in one of the following categories: teratoma, thymoma, thyroid, lymphoma (mediastinal tumor).
2. The radiology report describes hilar glands AND this is the only manifestation of PA/microbiologically proven sarcoidosis or tuberculosis (clarification: if there are other radiological findings consistent with sarcoidosis, label as “ILD”)

NB: if there is only a clinical suspicion of mediastinal tumor/hilar glands and/or a (PET-)CT or MRI showed signs mediastinal tumor/hilar glands, please refer this case to the Adjudication Committee.

**27. Thoracic pain of unknown origin**

Includes all patients with thoracic pain, in whom none of the aforementioned diagnostic labels is applicable, and for whom no other treatment was started except painkillers. May be indicated in the EHR as: “myogenic pain”, “costomyogenic pain”, “pain during extensive cough”.

Exceptions:

- Verify if the patient has visited the ED within 28 days after the first presentation with similar complaints. If so, please refer this case to the Adjudication Committee.
- If one or more of the other “… of unknown origin” labels is also applicable (i.e. “Dyspnea of unknown origin” and “Fever of unknown origin”), please refer this case to the Adjudication Committee.

**28. Dyspnea of unknown origin**

Includes all patients that have been discharged with shortness of breath, dyspnea or respiratory distress, in whom none of the aforementioned diagnostic labels is applicable.

Exceptions:

- Verify if the patient has visited the ED within 28 days after the first presentation with similar complaints. If so, please refer this case to the Adjudication Committee.
- If one or more of the other “… of unknown origin” labels is also applicable (i.e. “Thoracic pain of unknown origin” and “Fever of unknown origin”), please refer this case to the Adjudication Committee.
- Please refer hypoxemia without an explanatory cause to the Adjudication Committee.

Some examples of what may be indicated in the EHR:

- Hyperventilation is labelled "Dyspnea of unknown origin"
- Hyperventilation without dyspnea is labelled “Extrathoracic pathology"
- Hypoventilation syndrome related to obesity is labelled “Extrathoracic pathology”

**29. Fever of unknown origin**

Includes all patients with fever (temperature >38.0° Celsius), either measured at home or documented at the ED, without a focus. This can also be a single episode of documented fever.

Exceptions:

- Verify if the patient has visited the ED within 28 days after the first presentation with similar complaints. If so, please refer this case to the Adjudication Committee.
- If one or more of the other “… of unknown origin” labels is also applicable (i.e. “Thoracic pain of unknown origin” and “Dyspnea of unknown origin”), please refer this case to the Adjudication Committee.

Some examples of what may be indicated in the EHR:

- Sepsis without focus is labelled “Fever of unknown origin”
- Bacteremia or fever without focus are labelled “Fever of unknown origin”
- Sepsis due to a pneumonia is labelled “Pneumonia”
- Sepsis due to urinary tract infection is labelled “Extrathoracic pathology”
- For fever during chemotherapy and tumor fever, please also check the diagnostic labels “Extrathoracic pathology” and “No pathology” to decide which label is most applicable

**30. Other thoracic pathology**

Includes all thoracic diagnoses that are not mentioned in this handbook. Please specify the diagnosis in the open text field of the assessment form. Please keep in mind: this should be a clear diagnosis and not a list of symptoms or complaints. Everything below the diaphragm is considered extrathoracic pathology.

Some examples of what may be indicated in the EHR:

- Gastroesophageal reflux disease is labelled “Other thoracic pathology”
- Tietze syndrome is labelled “Other thoracic pathology”
- Exacerbation of, or new, bronchiectasis is labelled “Other thoracic pathology”
- Tako Tsubo cardiomyopathy is labelled “Other thoracic pathology”
- Renal asthma is labelled “Other thoracic pathology”
- Pathology of the stomach (i.e. peptic ulcer disease) is labelled “Extrathoracic pathology”

Exception:

- Please refer hemoptysis without an explanatory cause to the Adjudication Committee.

**31. Extrathoracic pathology**

Includes all extrathoracic diagnoses, where the diaphragm is considered the border between thorax and abdomen. Please specify the diagnosis in the open text field of the assessment form. Please keep in mind: this should be a clear diagnosis and not a list of symptoms or complaints.

This label can be assigned in two situations:

- This is the only diagnostic label for this patient
- This diagnostic label is combined with another label, but this diagnosis is relevant for the ED presentation

Please keep in mind: pre-existing extrathoracic pathology should not be labelled, except when the pre-existing condition worsened and is relevant for the ED presentation.

Some examples of what may be indicated in the EHR:

- Peptic ulcer disease is labelled “Extrathoracic pathology”
- CVC infection is labelled “Extrathoracic pathology”
- Systemic allergic reaction is labelled “Extrathoracic pathology”
- Panic attack is labelled “Extrathoracic pathology”
- Anemia is labelled “Extrathoracic pathology” only when the anemia contributes to the complaints of the patient at ED presentation. For example: when a patient experiences dyspnea with a hemoglobin level below 5 mmol/L. When a patient experiences a cough with a hemoglobin level below 5 mmol/L, the anemia does not cause the presenting symptom (cough) and thus should not be labelled.
- Tumor fever is labelled “Extrathoracic pathology” only when no antibiotics were given. In case antibiotic treatment was started, please label as “Fever of unknown origin”.

**32. No pathology**

Includes all patients that have been discharged without treatment and in whom none of the aforementioned diagnostic labels is applicable. If treatment has been started, please consider the options “Other thoracic pathology” or “Extrathoracic pathology”.

Some examples of what may be indicated in the EHR:

- An expected side effect of a medical procedure for which no treatment was started is labelled “No pathology”.
- Fever as a side effect of chemotherapy is labelled “No pathology” only if no antibiotics were given. If antibiotic treatment was started, this is considered “Fever of unknown origin”.

REFERENCES

1. Horan TC, Andrus M, Dudeck MA. CDC/NHSN surveillance definition of health care-associated infection and criteria for specific types of infections in the acute care setting. Am J Infect Control. 2008;36(5):309-32.

2. Jain S, Self WH, Wunderink RG, Fakhran S, Balk R, Bramley AM, et al. Community-Acquired Pneumonia Requiring Hospitalization among U.S. Adults. The New England journal of medicine. 2015;373(5):415-27.

3. Klein Klouwenberg PM, Ong DS, Bos LD, de Beer FM, van Hooijdonk RT, Huson MA, et al. Interobserver agreement of Centers for Disease Control and Prevention criteria for classifying infections in critically ill patients. Critical care medicine. 2013;41(10):2373-8.

4. Bird J, Biggs TC, Thomas M, Salib RJ. Adult acute rhinosinusitis. BMJ (Clinical research ed). 2013;346:f2687.

5. McCracken JL, Veeranki SP, Ameredes BT, Calhoun WJ. Diagnosis and Management of Asthma in Adults: A Review. Jama. 2017;318(3):279-90.

6. Vogelmeier CF, Criner GJ, Martinez FJ, Anzueto A, Barnes PJ, Bourbeau J, et al. Global Strategy for the Diagnosis, Management, and Prevention of Chronic Obstructive Lung Disease 2017 Report. GOLD Executive Summary. American journal of respiratory and critical care medicine. 2017;195(5):557-82.

7. Celli BR, MacNee W, Force AET. Standards for the diagnosis and treatment of patients with COPD: a summary of the ATS/ERS position paper. Eur Respir J. 2004;23(6):932-46.

8. De Boeck K, Wilschanski M, Castellani C, Taylor C, Cuppens H, Dodge J, et al. Cystic fibrosis: terminology and diagnostic algorithms. Thorax. 2006;61(7):627-35.

9. Rosenstengel A. Pleural infection-current diagnosis and management. J Thorac Dis. 2012;4(2):186-93.

10. Davies HE, Davies RJ, Davies CW, Group BTSPDG. Management of pleural infection in adults: British Thoracic Society Pleural Disease Guideline 2010. Thorax. 2010;65 Suppl 2:ii41-53.

11. MacDuff A, Arnold A, Harvey J, Group BTSPDG. Management of spontaneous pneumothorax: British Thoracic Society Pleural Disease Guideline 2010. Thorax. 2010;65 Suppl 2:ii18-31.

12. Bradley B, Branley HM, Egan JJ, Greaves MS, Hansell DM, Harrison NK, et al. Interstitial lung disease guideline: the British Thoracic Society in collaboration with the Thoracic Society of Australia and New Zealand and the Irish Thoracic Society. Thorax. 2008;63 Suppl 5:v1-58.

13. Glassberg J. Evidence-based management of sickle cell disease in the emergency department. Emerg Med Pract. 2011;13(8):1-20; quiz

14. Ballas SK, Lieff S, Benjamin LJ, Dampier CD, Heeney MM, Hoppe C, et al. Definitions of the phenotypic manifestations of sickle cell disease. Am J Hematol. 2010;85(1):6-13.

15. Damman P, van 't Hof AW, Ten Berg JM, Jukema JW, Appelman Y, Liem AH, et al. 2015 ESC guidelines for the management of acute coronary syndromes in patients presenting without persistent ST-segment elevation: comments from the Dutch ACS working group. Neth Heart J. 2017;25(3):181-5.

16. Ibanez B, James S, Agewall S, Antunes MJ, Bucciarelli-Ducci C, Bueno H, et al. 2017 ESC Guidelines for the management of acute myocardial infarction in patients presenting with ST-segment elevation: The Task Force for the management of acute myocardial infarction in patients presenting with ST-segment elevation of the European Society of Cardiology (ESC). Eur Heart J. 2018;39(2):119-77.

17. Genootschap NH. Stabiele angina pectoris [25-5-2018]. Available from: https://www.nhg.org/standaarden/samenvatting/stabiele-angina-pectoris.

18. Ponikowski P, Voors AA, Anker SD, Bueno H, Cleland JG, Coats AJ, et al. 2016 ESC Guidelines for the diagnosis and treatment of acute and chronic heart failure: The Task Force for the diagnosis and treatment of acute and chronic heart failure of the European Society of Cardiology (ESC). Developed with the special contribution of the Heart Failure Association (HFA) of the ESC. Eur J Heart Fail. 2016;18(8):891-975.

19. Hill SA, Booth RA, Santaguida PL, Don-Wauchope A, Brown JA, Oremus M, et al. Use of BNP and NT-proBNP for the diagnosis of heart failure in the emergency department: a systematic review of the evidence. Heart Fail Rev. 2014;19(4):421-38.

20. Adler Y, Charron P, Imazio M, Badano L, Baron-Esquivias G, Bogaert J, et al. 2015 ESC Guidelines for the diagnosis and management of pericardial diseases: The Task Force for the Diagnosis and Management of Pericardial Diseases of the European Society of Cardiology (ESC)Endorsed by: The European Association for Cardio-Thoracic Surgery (EACTS). Eur Heart J. 2015;36(42):2921-64.

21. Kruip MJ, Sohne M, Nijkeuter M, Kwakkel-Van Erp HM, Tick LW, Halkes SJ, et al. A simple diagnostic strategy in hospitalized patients with clinically suspected pulmonary embolism. Journal of internal medicine. 2006;260(5):459-66.

22. van der Hulle T, Cheung WY, Kooij S, Beenen LFM, van Bemmel T, van Es J, et al. Simplified diagnostic management of suspected pulmonary embolism (the YEARS study): a prospective, multicentre, cohort study. Lancet (London, England). 2017;390(10091):289-97.

23. Baldwin DR, White B, Schmidt-Hansen M, Champion AR, Melder AM, Guideline Development G. Diagnosis and treatment of lung cancer: summary of updated NICE guidance. BMJ (Clinical research ed). 2011;342:d2110.

**
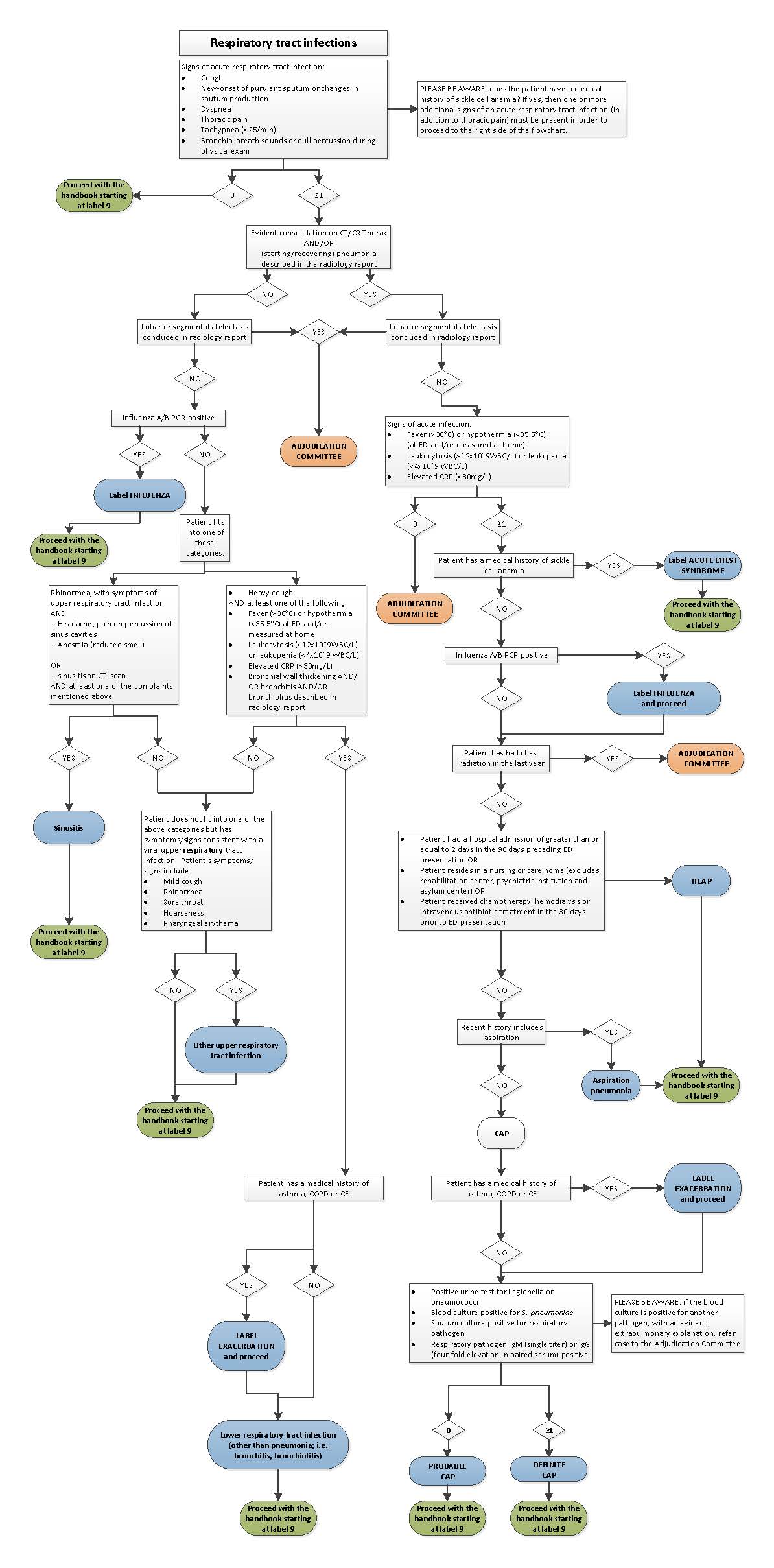
**

**Supplemental material 2.** Calculations of the reduction of working hours for medical specialists set against the hours of students and residents

A medical student spent 9.1 minutes (± 1.2 SD, range 7.5 to 10.7) on the independent assessment of a study participant. A resident spent on average 11.2 minutes on the assessment of a study participant. The discussion time per case during the consensus meeting of the students and the resident was on average 4.6 minutes.

Individual assessment of paper vignettes by a member of the expert panel took approximately 8 minutes per case. The discussion time per case during the consensus meeting of the medical specialists was 3.5 minutes.

Extrapolation to entire OPTIMACT cohort of 2,418 patients

Working hours medical students: 2,418 cases times 9.1 minute per case times two medical students equals 44,007.6 minutes of individual assessment. Based on this study, 45% of the study population is discussed during a consensus meeting with a resident: 45% of 2,418 cases equals 1,088 cases times 4.6 minutes discussion times two medical students equals 10,010.5 minutes. The total working hours of medical students equals 900 hours and 20 minutes.

Working hours residents: based on this study, 45% of the study population is assessed by residents. 45% of 2,418 cases equals 1,088 cases times 11.2 minutes individual assessment plus 4.6 minutes discussion time equals 17,190.4 minutes. The total working hours of residents equals 286 hours and 30 minutes.

Based on this study, 24% of the study population needs to be classified by medical experts.

Working hours medical specialists when all 2,418 patients were to be assessed by the expert panel (ergo: without the structured approach as presented in this study): all 2,418 cases would be assessed by the internist and pulmonologist: 2,418 cases times 8 minutes individual assessment times 2 experts (internist and pulmonologist) equals 38,688 minutes. Based on this study, 30% of these cases need to be assessed by the cardiologist as well (18 of the 60 cases in our validation study): 30% of 2,418 cases equals 725 cases times 8 minutes individual assessment times 1 expert (cardiologist) equals 5800 minutes. Based on this study, 43% of the cases is discussed during a consensus meeting of the expert panel (consisting of the internist, pulmonologist and chest radiologist): 43% of 2,418 cases equals 1,048 cases times 3.5 minutes discussion times 3 experts equals 11,004 minutes. The total working hours of medical specialists would equal 924 hours and 52 minutes. Working hours medical specialists when only 24% of the patients need to be assessed by the expert panel (ergo: with the structured approach as presented in this study). 24% of the abovementioned 924 hours and 52 minutes equals 221 hours and 58 minutes. The benefit would equal 702 hours and 54 minutes (924 hours and 52 minutes minus 221 hours and 58 minutes).

Conclusion

If the presented method is applied to the entire OPTIMACT study group of 2,418 patients, it is estimated to save approximately 703 hours of work by medical specialists. This has to be set against the (less expensive) hours of students and residents, which would approximate 900 hours and 286 hours, respectively.

**Supplemental material 3.** Reasons for disagreement between students

Of the 108 cases discussed in the consensus meeting between students and a resident, 76 were assigned a diagnosis. The reason for initial disagreement of the students was scored by the chair of the consensus meeting and categorized into other interpretation of the diagnostic handbook (31/76), information missed (e.g. culture result)(21/76), and different opinion on relevance of (extra) diagnostic label (e.g. whether a diagnostic label was relevant for this ED presentation versus considered a pre-existing diagnosis)(20/76). Four disagreements were due to procedural errors (e.g. error when filling in or processing the scoring form).

**Supplemental Table S1**. Inter-observer agreement between students for specific diagnostic labels in 240 cases

|  | Labels assigned  (*n* = 523) | Labels with agreement  (*n* = 334) | Concordance | Κ (95% CI) |
| --- | --- | --- | --- | --- |
| Definite diagnostic labels | 336 | 220 | 65% |  |
| Respiratory tract infections | 192 | 126 | 66% |  |
| Other LRTI | 70 | 34 | 49% | 0.43 (0.27-0.59) |
| CAP | 67 | 54 | 81% | 0.78 (0.66-0.89) |
| Influenza A/B | 28 | 22 | 79% | 0.77 (0.60-0.95) |
| HCAP | 14 | 8 | 57% | 0.56 (0.24-0.87) |
| URTI | 11 | 8 | 73% | 0.72 (0.42-1.02) |
| Aspiration pneumonia | 2 | 0 | 0% | - |
| Radiation pneumonitis | 0 | - | - | - |
| Other pulmonary diseases | 81 | 56 | 69% |  |
| Exacerbation COPD | 42 | 32 | 76% | 0.74 (0.59-0.89) |
| Exacerbation asthma | 30 | 22 | 73% | 0.72 (0.53-0.90) |
| ILD | 4 | 0 | 0% | - |
| Pneumothorax | 3 | 2 | 67% | 0.67 (0.05-1.28) |
| Pleural effusion or empyema | 1 | 0 | 0% | - |
| Pleuritis sicca | 1 | 0 | 0% | - |
| Acute Chest Syndrome | 0 | - | - | - |
| Atelectasis | 0 | - | - | - |
| Exacerbation CF | 0 | - | - | - |
| Heart diseases | 49 | 32 | 65% |  |
| ACS | 18 | 14 | 78% | 0.77 (0.55-0.99) |
| Cardiac failure | 15 | 8 | 53% | 0.52 (0.21-0.83) |
| Cardiac arrhythmias | 12 | 8 | 67% | 0.66 (0.34-0.97) |
| Pericarditis | 3 | 2 | 67% | 0.67 (0.05-1.28) |
| Stable angina pectoris | 1 | 0 | 0% | - |
| Vascular disease | 10 | 6 | 60% |  |
| Pulmonary embolism | 10 | 6 | 60% | 0.59 (0.23-0.96) |
| Nodules and tumors | 4 | 0 | 0% |  |
| Lung cancer and pulmonary metastases | 4 | 0 | 0% | - |
| Mediastinal tumor | 0 | - | - | - |
| Other diagnostic labels | 187 | 114 | 61% |  |
| Extrathoracic pathology | 101 | 68 | 67% | 0.59 (0.46-0.71) |
| Thoracic pain of unknown origin | 51 | 42 | 82% | 0.80 (0.68-0.93) |
| Fever of unknown origin | 12 | 2 | 17% | 0.15 (-0.14-0.44) |
| Other thoracic pathology | 12 | 0 | 0% | - |
| Dyspnea of unknown origin | 11 | 2 | 18% | 0.17 (-0.14-0.47) |
| No pathology | 0 | - | - | - |
| Direct referral to the Adjudication Committee | 89 | 50 | 56% |  |

Data represent inter-observer agreement between medical students. ACS = acute coronary syndrome, CAP = community-acquired pneumonia, COPD = chronic obstructive pulmonary disease, HCAP = healthcare-associated pneumonia, HCAP = healthcare-associated pneumonia, ILD = interstitial lung disease, LRTI = lower respiratory tract infection, URTI = upper respiratory tract infection. Within diagnostic categories, rows are sorted first by the number of labels assigned and then alphabetically.

**Supplemental Table S2**. Classification by the expert panel in 60 validation cases

| Diagnostic label(s) internist | Diagnostic labels(s) pulmonologist | Diagnostic label(s) cardiologist | Number of cases |
| --- | --- | --- | --- |
| Agreement |  |  | 24 |
| Single label cases |  |  | 17 |
| Extrathoracic pathology | Extrathoracic pathology |  | 8 |
| CAP | CAP |  | 4 |
| Thoracic pain of unknown origin | Thoracic pain of unknown origin | Thoracic pain of unknown origin | 3 |
| Exacerbation COPD | Exacerbation COPD |  | 1 |
| Other LRTI | Other LRTI |  | 1 |
| Multiple label cases |  |  | 7 |
| CAP, influenza A/B | CAP, influenza A/B |  | 2 |
| Other LRTI, exacerbation asthma | Other LRTI, exacerbation asthma |  | 2 |
| CAP, exacerbation COPD | CAP, exacerbation COPD |  | 1 |
| CAP, ILD | CAP, ILD |  | 1 |
| Influenza A/B, exacerbation asthma | Influenza A/B, exacerbation asthma |  | 1 |
| Partial agreement |  |  | 23 |
| Multiple label cases |  |  | 23 |
| HCAP | HCAP, extrathoracic pathology* |  | 2 |
| CAP | CAP, cardiac arrhythmia | CAP, cardiac arrhythmia | 1 |
| CAP | CAP, exacerbation COPD |  | 1 |
| CAP | CAP, other thoracic pathology |  | 1 |
| CAP, exacerbation asthma, exacerbation COPD | CAP, exacerbation asthma, exacerbation COPD, exacerbation CF* |  | 1 |
| CAP, influenza A/B | CAP, influenza A/B, exacerbation CF* |  | 1 |
| Cardiac arrhythmia, thoracic pain of unknown origin | Cardiac arrhythmia, thoracic pain of unknown origin | Cardiac arrhythmia | 1 |
| Cardiac failure, other LRTI | Cardiac failure | Cardiac failure, sinusitis, cardiac arrhythmia | 1 |
| Exacerbation asthma | Exacerbation asthma, thoracic pain of unknown origin* | Exacerbation asthma, thoracic pain of unknown origin* | 1 |
| Exacerbation COPD | Exacerbation COPD, cardiac failure | Exacerbation COPD, cardiac failure | 1 |
| Extrathoracic pathology | Extrathoracic pathology, other LRTI |  | 1 |
| Fever of unknown origin | Fever of unknown origin, extrathoracic pathology* |  | 1 |
| Influenza A/B | Influenza A/B, cardiac failure | Influenza A/B | 1 |
| Influenza A/B | Influenza A/B, extrathoracic pathology* |  | 1 |
| Influenza A/B | Influenza A/B, other LRTI* |  | 1 |
| Influenza A/B, exacerbation COPD | Influenza A/B, exacerbation COPD, ACS with elevated troponin levels | Influenza A/B, exacerbation COPD | 1 |
| Influenza A/B, other thoracic pathology | Influenza A/B, other thoracic pathology, exacerbation COPD |  | 1 |
| Other LRTI, exacerbation COPD | Exacerbation COPD |  | 1 |
| Sinusitis, exacerbation asthma | Exacerbation asthma |  | 1 |
| Thoracic pain of unknown origin | Thoracic pain of unknown origin, exacerbation CF* | Thoracic pain of unknown origin | 1 |
| Thoracic pain of unknown origin | Thoracic pain of unknown origin, exacerbation COPD | Thoracic pain of unknown origin | 1 |
| URTI | URTI, exacerbation COPD |  | 1 |
| Disagreement |  |  | 13 |
| Single label cases |  |  | 6 |
| Extrathoracic pathology | Fever of unknown origin |  | 2 |
| AC | Pulmonary embolism |  | 1 |
| ACS with elevated troponin levels | ACS with elevated troponin levels | Other thoracic pathology | 1 |
| Fever of unknown origin | Extrathoracic pathology* |  | 1 |
| Thoracic pain of unknown origin* | Extrathoracic pathology* | Thoracic pain of unknown origin* | 1 |
| Multiple label cases |  |  | 7 |
| AC | Aspiration pneumonia, extrathoracic pathology |  | 1 |
| AC | CAP, influenza A/B |  | 1 |
| AC | Influenza A/B, cardiac failure | Influenza A/B | 1 |
| AC | Other thoracic pathology, extrathoracic pathology |  | 1 |
| Cardiac failure | Cardiac failure, cardiac arrhythmia | Cardiac arrhythmia | 1 |
| Thoracic pain of unknown origin | Thoracic pain of unknown origin, other thoracic pathology | Other thoracic pathology | 1 |
| Other LRTI | URTI, exacerbation asthma |  | 1 |

Data represent classification by two members of the expert panel: the internist and the pulmonologist. *Cases where at least one cardiologic label was assigned or the additional diagnostic category ‘thoracic pain of unknown origin’ or ‘other thoracic pathology’ was assigned, were also classified by the cardiologist. Abbreviations: AC = adjudication committee, ACS = acute coronary syndrome, CAP = community-acquired pneumonia, CF = cystic fibrosis, COPD = chronic obstructive pulmonary disease, HCAP = healthcare-associated pneumonia, ILD = interstitial lung disease, LRTI = lower respiratory tract infection, URTI = upper respiratory tract infection. Within categories of agreement, rows are sorted first by prevalence and then alphabetically. *Disagreement on the diagnosis was based on discordance on a procedural error or labels from the additional diagnostic categories only. These are considered agreement cases.

**Supplemental Table S4**. Inter-observer agreement between members of the expert panel for specific diagnostic labels in 60 validation cases

|  | Labels assigned  (*n* = 173) | Labels with agreement  (*n* = 142) | Concordance | Κ (95% CI) |
| --- | --- | --- | --- | --- |
| Definite diagnostic labels | 119 | 102 | 86% |  |
| Respiratory tract infections | 71 | 64 | 90% |  |
| CAP | 28 | 28 | 100% | 1.00 (1.00-1.00) |
| Influenza A/B | 22 | 22 | 100% | 1.00 (1.00-1.00) |
| Other LRTI | 11 | 6 | 55% | 0.50 (0.12-0.88) |
| HCAP | 4 | 4 | 100% | 1.00 (1.00-1.00) |
| URTI | 4 | 2 | 50% | 0.48 (-0.13-1.10) |
| Aspiration pneumonia | 2 | 2 | 100% | 1.00 (1.00-1.00) |
| Radiation pneumonitis | 0 | - | - | - |
| Other pulmonary diseases | 31 | 26 | 84% |  |
| Exacerbation COPD | 16 | 12 | 75% | 0.71 (0.46-0.97) |
| Exacerbation asthma | 13 | 12 | 92% | 0.91 (0.75-1.08) |
| Acute Chest Syndrome | 0 | - | - | - |
| Atelectasis | 0 | - | - | - |
| Exacerbation CF | 0 | - | - | - |
| ILD | 2 | 2 | 100% | 1.00 (1.00-1.00) |
| Pleural effusion or empyema | 0 | - | - | - |
| Pleuritis sicca | 0 | - | - | - |
| Pneumothorax | 0 | - | - | - |
| Heart diseases | 15 | 10 | 67% |  |
| Cardiac failure* | 8 | 6 | 75% | 0.73 (0.38-1.08) |
| Cardiac arrhythmias* | 4 | 2 | 50% | 0.49 (-0.11-1.09) |
| ACS* | 3 | 2 | 67% | 0.66 (0.04-1.28) |
| Pericarditis | 0 | - | - | - |
| Stable angina pectoris | 0 | - | - | - |
| Vascular disease | 2 | 2 | 100% |  |
| Pulmonary embolism | 2 | 2 | 100% | 1.00 (1.00-1.00) |
| Nodules and tumors | 0 | - | - |  |
| Lung cancer and pulmonary metastases | 0 | - | - | - |
| Mediastinal tumor | 0 | - | - | - |
| Other diagnostic labels | 57 | 40 | 70% |  |
| Extrathoracic pathology | 30 | 22 | 73% | 0.65 (0.43-0.87) |
| Thoracic pain of unknown origin* | 16 | 14 | 88% | 0.86 (0.66-1.05) |
| Fever of unknown origin | 5 | 2 | 40% | 0.38 (-0.18-0.93) |
| Other thoracic pathology* | 5 | 2 | 40% | 0.38 (-0.15-0.92) |
| Dyspnea of unknown origin | 1 | 0 | 0% | - |
| No pathology | 0 | - | - | - |

Data represent inter-observer agreement for two members of the expert panel: the internist and the pulmonologist. *Cases where at least one cardiologic label was assigned or the additional diagnostic category ‘thoracic pain of unknown origin’ or ‘other thoracic pathology’ was assigned, were also assessed by the cardiologist prior to the plenary expert meeting. ACS = acute coronary syndrome, CAP = community-acquired pneumonia, CF = cystic fibrosis, COPD = chronic obstructive pulmonary disease, HCAP = healthcare-associated pneumonia, HCAP = healthcare-associated pneumonia, ILD = interstitial lung disease, LRTI = lower respiratory tract infection, URTI = upper respiratory tract infection. Within diagnostic categories, rows are sorted first by the number of labels assigned and then alphabetically.
